# Advance of Microemulsion and Application for Enhanced Oil Recovery

**DOI:** 10.3390/nano14121004

**Published:** 2024-06-10

**Authors:** Kaiqi Leng, Baoshan Guan, Weidong Liu, Chen Jiang, Sunan Cong, Baoliang Peng, Yufan Tao

**Affiliations:** 1 School of Engineering Sciences, University of Chinese Academy of Sciences, Beijing 100049, China; lengkaiqi20@mails.ucas.ac.cn (K.L.); lwd69@petrochina.com.cn (W.L.); jiangchen98@petrochina.com.cn (C.J.); taoyufan21@petrochina.com.cn (Y.T.); 2 Institute of Porous Flow and Fluid Mechanics, Chinese Academy of Sciences, Langfang 065007, China; 3 Research Institute of Petroleum Exploration and Development, PetroChina, Beijing 100083, China; csn69@petrochina.com.cn (S.C.); pengbl@petrochina.com.cn (B.P.); 4 Key Laboratory of Nano Chemistry (KLNC), CNPC, Beijing 100083, China

**Keywords:** microemulsion, enhanced oil recovery, influence factor, remaining oil characterization

## Abstract

With the ongoing advancement in oil exploration, microemulsion, as an innovative oil displacement method, has garnered considerable attention owing to its exceptional physicochemical properties in enhancing crude oil recovery. As such, this study initially delineates the fundamental concepts, classifications, formation mechanisms, advantages, and preparation methodologies of microemulsions. Subsequently, it introduces the selection criteria for microemulsion components, followed by an elucidation of the characterization methods for microemulsions based on these criteria. Furthermore, it examines the factors influencing the efficacy of microemulsions in enhancing oil recovery through two distinct methods, along with the effects of various formulation microemulsions under laboratory and oilfield conditions. Additionally, it outlines prospects, challenges, and future development trends pertaining to microemulsions.

## 1. Introduction

**Figure 1:**
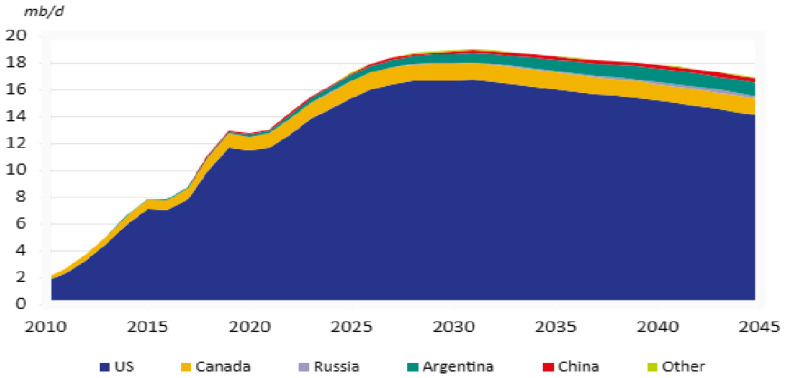
World tight oil development [6] (data source: OPEC).

Energy serves as the cornerstone of human societal development and constitutes the indispensable material foundation for national economic endeavors [1]. With the rapid advancement in the global economy, the demand for energy continues to escalate, while the available resources for development and utilization steadily decline. In response to the global energy crisis, numerous nations are actively pursuing alternative energy sources such as unconventional oil and unconventional natural gas [2]. Among these, tight oil emerges as a critically significant unconventional petroleum resource, bridging the gap between conventional oil and shale gas. Characterized by extensive distribution and substantial reserves, tight oil resources play a pivotal role in various regions, including the United States, Canada, Russia, and several European countries, where they contribute to approximately 20% to 30% of the total resource potential [3]. The resource potential of tight oil is enormous, with discovered reserves exceeding 1 billion tons and widespread distribution, characterized by a substantial scale of reserves. Compared with conventional petroleum, tight oil boasts a shorter development time, higher recovery rate, and faster oil recovery speed [4]. Therefore, the development of tight oil represents a beneficial exploration of new types of unconventional oil and gas resources. According to statistics, the global recoverable reserves of tight oil are approximately 472.8 × 10^8^ t, primarily concentrated in countries such as the United States, Canada, and Argentina [5]. According to OPEC data predictions, as illustrated in Figure 1, the future global supply of tight oil will remain dominated by the United States while other countries maintain moderate levels. It is estimated that by 2025, global tight oil production will increase to 1540 × 10^4^ bbl/d [6].

The successful development of tight oil in North America offers valuable insights for our country. However, because of geological variations, further in-depth research into tight oil development in China is necessary. Chemical flooding remains the primary technology for enhancing oil recovery in onshore heterogeneous reservoirs in our country, which leads globally in this field. With its mature conceptual framework and supporting technologies, chemical flooding has achieved the stable production of tens of millions of tons annually for 21 consecutive years, with a cumulative oil production of 280 million tons [7]. Nevertheless, as oilfield grades decline and the development of tight oilfields progresses, the challenges in further oil extraction intensify. Given China’s status as a major energy consumer, accelerating oilfield development and increasing domestic oil production are imperative to alleviate the country’s high dependence on foreign energy sources. Therefore, addressing such challenges requires urgent exploration of new oil recovery methods in Chinese oilfields to enhance production and meet domestic crude oil demand.

Microemulsion, as a significant branch of nanotechnology for oil recovery, exhibits evident advantages. It is a colloidal dispersion system formed spontaneously under certain conditions by the action of surfactants and cosurfactants on oil and water, differing from traditional nanoemulsions. Microemulsions represent a thermodynamically stable system [8]. Research indicates that microemulsions play a crucial role as oilfield displacement agents by dissolving colloids and asphaltenes, increasing fracture half-length, reducing water phase trapping, restoring relative reservoir permeability, and forming oil barriers [9]. Microemulsions, as a chemical flooding technique akin to mixed-phase displacement, offer numerous advantages. Firstly, they are not restricted by water content, allowing for high ultimate recovery rates. Secondly, they possess high surface activity, reducing interfacial tension with crude oil to as low as 10^−2^ to 10^−3^ mN/m. Thirdly, and most importantly, they exhibit small particle sizes, with droplet radii ranging from 5 nm to 500 nm. Considering these advantages, microemulsions serve as effective displacement agents for enhancing recovery rates in low-permeability reservoirs.

Currently, research on microemulsions predominantly remains at the theoretical and laboratory experimentation stages. Therefore, in addition to providing detailed explanations on the formation mechanism of microemulsions, this paper also summarizes their recent field applications in oilfields, including various injection methods. This sets the foundation for selecting suitable systems and injection methods for different reservoirs. Finally, it offers insight into the future development trends of microemulsions in enhancing oil recovery.

## 2. Research on the Introduction, Types, and Formation Mechanisms of Microemulsions

### 2.1. Profile and Types of Microemulsions

**Figure 2:**
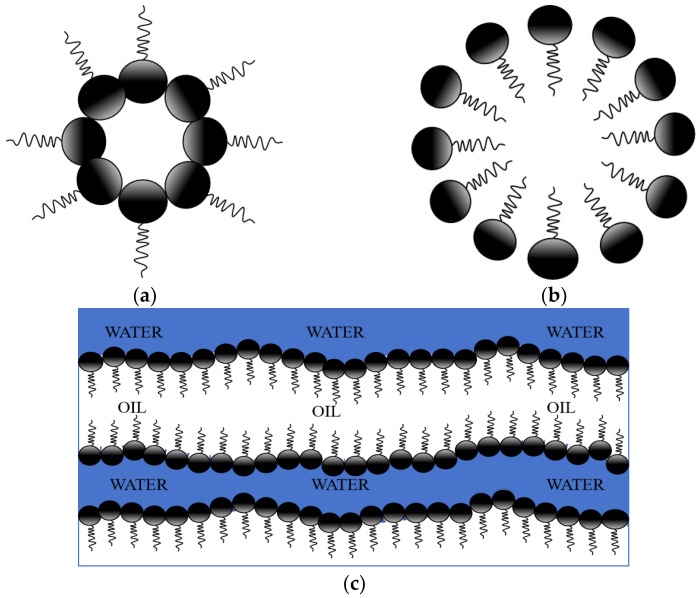
Schematic diagram of different types of microemulsion [12]: (**a**) W/O type; (**b**) O/W type; (**c**) bicontinuous type.

**Figure 3:**
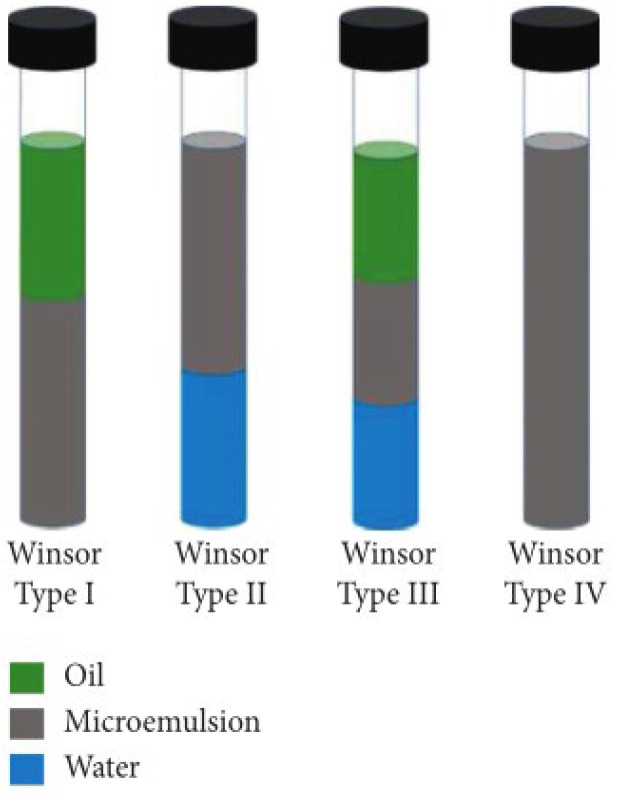
Schematic diagram of different types of microemulsions (by phase) [13].

Microemulsions were first defined by Schulamn, in 1959, as transparent or translucent mixed-phase systems composed of surfactants, cosurfactants, organic solvents, and water. They can spontaneously form without the need for external energy input [10]. The formation mechanisms of microemulsions are diverse, and research methods in this regard continue to expand. Based on their equilibrium states, microemulsions can be classified into multiphase and single-phase categories. Single-phase microemulsions (Winsor Type IV) do not contain excess oil and/or water; they consist of homogeneously dispersed oil-in-water (O/W) or water-in-oil (W/O) microemulsions. Multiphase microemulsions include Winsor Type I (O/W microemulsions coexist with excess oil phase), Winsor Type II (W/O microemulsions coexist with excess water phase), and Winsor Type III (bicontinuous microemulsions coexist with excess oil and water phases). The actual application systems have relatively small ranges within the relative oil and water regions, including single-phase W/O and O/W microemulsions of Winsor Type IV, as well as bicontinuous microemulsions of Winsor Type III [11], as illustrated in Figure 2 and Figure 3.

**Figure 4:**
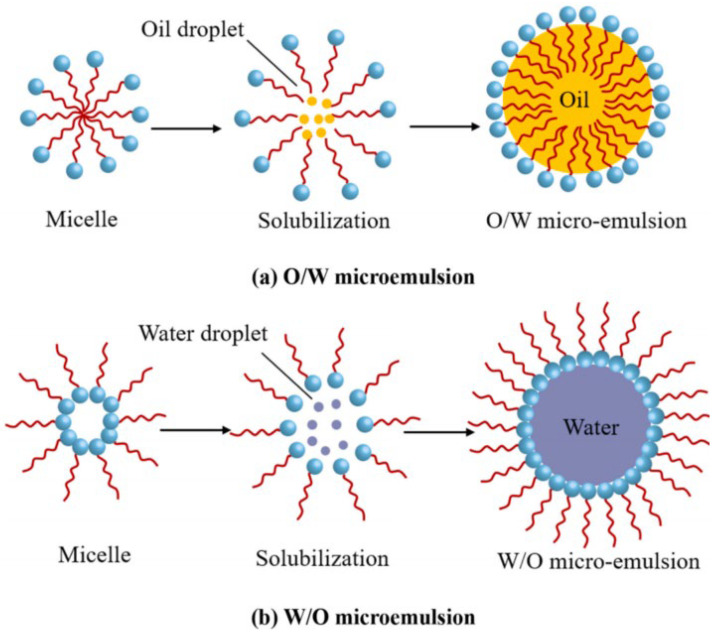
Formation process of microemulsions [8].

During the development of microemulsions, various formation mechanisms have been proposed, including the instantaneous reduction in interfacial tension theory, interfacial adsorption film theory, R ratio theory, and micelle solubilization theory. The formation process of microemulsions shares some similarities with micelle solubilization [14]. This process is illustrated in Figure 4.

Microemulsions can be formed not only by directly adding additives and other components to surfactants but also by adding alkaline water to crude oil, which undergoes chemical reactions to generate microemulsions in situ within the reservoir. The performances of these in situ microemulsions depends on factors such as the salinity, type, and concentration of the alkaline solution, as well as the pH of the system, total acid number (TAN), and equivalent alkane carbon number (EACN) of the crude oil [15]. For instance, in low-salinity environments, in situ microemulsions formed in low-molecular-weight linear hydrocarbons can exhibit bicontinuous regions (middle-phase microemulsions) [16]. As previously discussed, microemulsions can be categorized into three types, yet these types can undergo phase transitions based on the salinity of the aqueous phase [17]. With increasing salinity, microemulsions transition to inverse microemulsions. At low salinity levels, the system exhibits an O/W phase, but as salinity increases, the system transitions to a bicontinuous phase, ultimately forming W/O type microemulsions [12].

Microemulsions are not only formed by synthetic surfactants. After adding alkaline water, the surfactant mixture can be formed in situ by reacting with the acidic components in the oil, and the acidic components can form microemulsions in principle. The performance of the surfactant mixture depends on the salinity, type, and concentration of the alkaline solution; the pH value of the system; and the total acid number (TAN) and equivalent alkane carbon number (EACN) of the crude oil [15]. The surfactant mixture formed in low-molecular-weight linear hydrocarbons forms a bicontinuous region (microemulsion) in a low-salinity environment [16]. However, for different types of crude oil, the salinity range is affected by EACN, thus forming microemulsion phase [17]. The structure of microemulsion depends on salinity, water cut, cosurfactant concentration and surfactant concentration. When the water cut is high, the microemulsion is an O/W system, and the oil is dissolved in the micelle core. Although the mixture remains single-phase and thermodynamically stable, the structure of the microemulsion changes through a series of intermediate states. In addition, the salinity of the aqueous phase can also change the structure of the microemulsion. As the salinity increases, the microemulsion changes to a reverse microemulsion. At low salinity, the system is O/W phase, but with the increase in salinity, the system changes into W/O microemulsion [12].

### 2.2. Characteristics of Microemulsion

Microemulsions are thermodynamically stable, optically isotropic liquid systems formed by multiple components, and their properties differ from other emulsions, as shown in Table 1. Microemulsions have diverse characteristics and can be categorized into the following five types: (a) Microemulsions have small particle sizes, often in the nanometer range, and exist as transparent or translucent clear solutions, exhibiting strong fluidity. Upon solvent addition, they are diluted and rapidly dispersed, demonstrating excellent permeability. This facilitates their penetration into the tiny pores of reservoirs, enhancing the efficiency of contact between the treating fluid and the formation. (b) Microemulsions exhibit ultralow interfacial tension due to the presence of a significant amount of surfactants. Upon entering reservoir pores, they disperse within the solution, with the dissolved surfactants dispersing more rapidly in the fluid region compared to individual surfactants. This enables them to be transported to solid–liquid and liquid–liquid interfaces more quickly, resulting in faster action. Microemulsions typically have surface tensions below 30 mN·m, with interfacial tensions ranging from 10^−2^ to 10^−3^ mN/m. If the surfactant performance is excellent, interfacial tensions can decrease to 10^−4^ mN/m. (c) Microemulsions effectively alter rock wettability, reducing the adsorption energy of crude oil on the rock surface, leading to the detachment of oil droplets adhered to the rock surface. (d) Microemulsions exhibit strong solubilization capabilities, being able to dissolve water in any proportion and possessing high dissolution power. They can dissolve various substances, such as asphaltenes, waxes, gel cakes, wellbore debris, and formation particles, demonstrating an oil-washing effect and alleviating formation channel blockage phenomena. (e) Microemulsions are thermodynamically stable; they do not undergo phase separation or demulsification even after prolonged periods of storage and centrifugation. Their single-phase states remain stable in highly diluted oil and water phases. Even when added to solutions at concentrations of 0.1% to 0.5%, they maintain stability [18,19].

With the reduction in the complexity and preparation limitations of microemulsions, their ultralow interfacial tension, large interfacial area, thermodynamic stability, and high solubility in immiscible solutions make them increasingly significant in science and industry. They cover a variety of areas, including alternative fuels, contaminated soil remediation, drug delivery, nanoparticle synthesis, agrochemicals, food, cosmetics, and chemical flooding for enhanced oil recovery [20].

**Table 1:** Comparison of the properties of different emulsions [21].

Emulsion Type	Macroemulsion	Nanoemulsion	Microemulsion	Swelling Micelle Solution
Particle size	1~10 μm	20~500 nm	10~100 nm	<50 nm
Emulsifier concentration	Low	Middle	High	More than the critical micelle concentration
Appearance	Opacity	Transparent, translucent, or milky white	Transparent, translucent	Transparent
Stability	Instability	Long-term dynamic stability	Thermodynamic stability	Thermodynamic stability
Type	O/W and W/O	O/W and W/O	O/W, W/O, and bicontinuous structures	Micelle or reverse micelle solution

### 2.3. Formation Mechanisms of Microemulsion

As previously mentioned, various formation mechanisms have been proposed during the development of microemulsions. These primarily include the instantaneous reduction in interfacial tension theory, interfacial adsorption film theory, R ratio theory, and micelle solubilization theory.

(1) Instantaneous negative interfacial tension theory

This theory, jointly proposed by Schulman and Prince on the basis of the Gibbs equation, suggests that as the number of active components in the system increases, more molecules are likely to undergo mixed adsorption at the interface, leading to further reduction in interfacial tension (IFT). Therefore, through the combined action of surfactants and cosolvents, ultralow interfacial tension, and even instantaneous negative interfacial tension (IFT < 0) can be achieved. Because of the instability of negative interfacial tension, the oil–water interface spontaneously expands. More active molecules are adsorbed at the interface, causing the interfacial tension to return to a positive value. The spontaneous expansion of the interface promotes the formation of microemulsions. If the droplets in the microemulsion coalesce, the reduction in the interfacial area may even lead to negative interfacial tension, promoting the expansion of the interface to resist droplet coalescence. This also explains the thermodynamic stability of microemulsions [22]. However, the limitation of this theory lies in the inability to measure negative interfacial tension values in laboratory settings, restricting research to the theoretical stage.

(2) Dual-membrane theory

**Figure 5:**
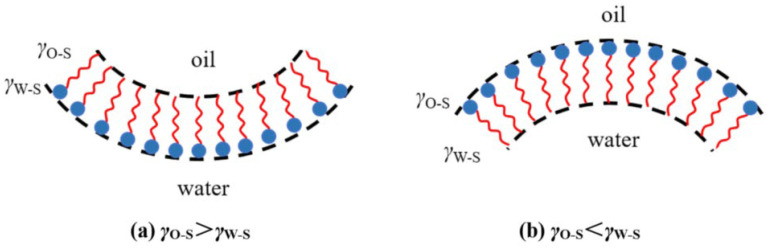
Mechanism of the facial mask at the adsorption interface [8].

This theory, primarily represented by Schulman and Bowcott, suggests that surfactants and cosolvents in microemulsions can form interfacial adsorption films of a certain thickness, which can be considered as a third phase. The two sides of this film are in contact with the aqueous phase and the oil phase, respectively. The interaction between the film and the oil phase or water phase affects its bending direction, thereby determining the type of microemulsion [23,24]. Assuming the interfacial tensions between the film and the oil phase and water phase are $\gamma_{OS}$ and $\gamma_{WS}$, respectively, when $\gamma_{OS}$ > $\gamma_{WS}$, the thin film bends toward the oil phase until equilibrium is reached, forming an O/W microemulsion; when $\gamma_{OS}$ < $\gamma_{WS}$, the film bends toward the water phase, forming a W/O type microemulsion, as shown in Figure 5.

(3) R-ratio theory

The R-ratio theory is the most mature theory in the formation of microemulsions, primarily studying the interactions among molecules. Surfactants, as amphiphilic molecules, interact simultaneously with oil and water, and the superposition of these interactions determines the preferred bending direction of the interface film. This theory describes the interactions among surfactants, cosolvents, the aqueous phase, and the oil phase [25], as shown in the following Equation (1):

(1) $$R=\frac{A_{SO}-A_{OO}-A_{ii}}{A_{SW}-A_{WW}-A_{hh}}$$

where $A_{SO}$ and $A_{SW}$ are the cohesive energies between the oil/surfactant and water/surfactant, respectively; $A_{SW}$ and $A_{WW}$ are the cohesive energies between oil molecules and water molecules; $A_{\mathrm{ii}}$ is the cohesive energy between the hydrophobic groups of surfactants; and $A_{hh}$ is the cohesive energy between the head groups of the surfactants.

When *R* = 1, the interface does not bend, forming a bicontinuous structure; when *R* < 1, the force between the interface and the oil phase is stronger. To achieve equilibrium, the interface bends toward the water phase, forming an O/W microemulsion; when *R* > 1, it is easier to form a W/O microemulsion. The R-ratio actually reflects the strength of the hydrophilic–hydrophobic nature of the interface layer, which contributes to the changes in the microemulsion structure. According to this theory, the adjustment of the R-ratio can be achieved by increasing the branching or chain length of the hydrophobic groups in the surfactant, thus enabling the transformation of microemulsion types [8].

(4) Micelle solubilization theory

Solubilization refers to the process in which poorly soluble substances, under the action of surfactants, increase their solubility in a solvent and form a solution [26]. Many researchers believe that microemulsions share many similarities with micellar solutions, to the extent that some consider microemulsions to be the result of micelle solubilization. When the concentration of surfactants exceeds their critical micelle concentration (CMC), the surfactants spontaneously form micelles with strong solubilization capabilities.

(5) Geometric arrangement theory

Building upon the foundation of the interfacial adsorption film theory, scholars have combined molecular arrangement theories to establish molecular arrangement models of interface films, which can clearly illustrate bending directions. By introducing a packing parameter, §, to characterize the arrangement of molecules at the interface. The expression for *P* is given by the following:

(2) $$P=\frac{V}{a_{0}I_{C}}$$

where $a_{0}$ is the equilibrium area of the surfactant head group, and $l_{c}$ and *V* are the length and volume of the surfactant hydrophobic chain, respectively; when *P* > 1, the interface film points to the oil phase, which is beneficial to the formation of the W/O microemulsion. When 1/3 < *P* < 1, this is an essential condition for the formation of the O/W microemulsion. When *P* < 1/3, no microemulsion is formed, but micelles are formed [27].

### 2.4. Research on the Components of Microemulsion

It is well known that microemulsions are composed of surfactant, cosurfactant, oil phase, and a water phase or salt. The different contents, concentrations, structures, and formulations of these five components will result in different physical and chemical properties of microemulsions, and the role in the application process is also different. Therefore, it is necessary to select the appropriate components for the specific formation to obtain a stable microemulsion system. The effects of each component on the microemulsion system are described below.

#### 2.4.1. Effect of Surfactant Selection on the Properties of Microemulsion

As the most important component of the microemulsion system, it is necessary to make appropriate choices for surfactants. Surfactants can be generally divided into the following four types: anionic surfactants, cationic surfactants, zwitterionic surfactants, and nonionic surfactants.

The anionic surfactant is one of the earliest surfactant industry developments, with the largest production, the most varieties, and the most mature industrialization. Among the anionic surfactants, the sulfonate type has the largest yield and is the most widely used, followed by the sulfate type [28,29]. In general, it has poor compatibility with cations [30]. When soluble with anionic surfactants, it can greatly improve the physical and chemical properties of both [31]. Cationic surfactants have excellent bactericidal properties, but they are also highly toxic and have poor biodegradability [32]. They are easily adsorbed on solid surfaces and easily block pores during formation. Therefore, cationic surfactants are, generally, not applied alone in oilfield development [33]. Amphoteric surfactants have excellent performance [34], good compatibility with anionic, cationic, and nonionic surfactants [35,36,37], and good acid and alkali resistance [38], but the production cost is expensive, so the yield is low [39]. A nonionic surfactant has excellent chemical properties. Its production is second only to anionic surfactants [40], and it does not ionize in water. Therefore, it does not easily adsorb to a great degree on a rock surface [41], but it forms hydrogen bonds with water, which makes its properties more stable [42]. It is not affected by acid, alkali, and salt, and it has strong hard water resistance [43]. Because a nonionic surfactant has good compatibility with the other three types of surfactants, it can be compounded with them when used [44,45], and a nonionic surfactant has high surface activity and strong solubilization [46]. It has a good effect on oilfield development.

When selecting surfactants, it is also important to consider the physicochemical properties of the surfactants. Firstly, the structure of the surfactant has a significant impact on the formation of microemulsions. Zhang et al. studied the impact of surfactant branching on microemulsion formation through mesoscale simulations and dissipative dynamics. The results indicate that under the same surfactant concentration, linear surfactants are more likely to form microemulsions, and, at this point, the interfacial tension is at its lowest [47]. Additionally, the hydrophilic–lipophilic balance (HLB) of the surfactant also influences the formation of microemulsion phases. Yuwanti et al. selected three surfactants with low, medium, and high HLB values to prepare microemulsions. The surfactant with a medium HLB value was able to connect the oil and water phases with the surfactant layer, increasing the interaction between the surfactant–oil and surfactant–water, thus resulting in a more stable O/W microemulsion [48]. Subsequently, the interfacial tension of the surfactant can also affect the formation of microemulsions. Zhou et al. tested the interfacial tension of DPSB at various concentrations and found that the microemulsion volume was maximized at the concentration corresponding to the lowest interfacial tension [49].

In addition to the structure, HLB value, and interfacial tension, the critical micelle concentration (CMC) of the surfactant also significantly influences the ease of microemulsion formation. Li et al. tested the surface tension of several different types of surfactants and found that nonionic surfactants with lower CMC have a stronger self-aggregation capability in solution. This results in enhanced solubilization capacity, making substances that are insoluble or sparingly soluble in water more likely to enter micelles and significantly increase their solubility. Consequently, the formed microemulsions exhibit high permeability and wetting properties [50]. Vo and colleagues arrived at similar conclusions, and leveraging the low toxicity and ready biodegradability of APG (alkyl polyglycoside), they discussed the potential for plant-derived green surfactants like APG to form microemulsions [51]. Aveyard et al. also discussed the impact of different types of surfactants on microemulsion formation. The results indicated that for nonionic surfactants, as the temperature increases, the amount of oil phase adsorbed on the interfacial membrane progressively decreases, but at high concentrations, the adsorption increases, which favors the formation of microemulsions. In contrast, the trend for anionic surfactants is exactly opposite to that of nonionic surfactants. At low concentrations, they are beneficial for the formation of microemulsions and also significantly contribute to the reduction in system costs [52]. Wu et al. investigated the synergistic effects of anionic-nonionic mixed surfactants and found that the CMC (critical micelle concentration) lies between the values for the individual surfactants. As the concentration of the nonionic surfactant increases, the absolute value of the synergistic interaction increases, and the likelihood of spontaneous mixed micelle formation also increases [53].

Therefore, when selecting surfactants, one must consider aspects such as the type of surfactant and its physicochemical properties. Table 2 provides a reference for the types of microemulsion systems prepared from different surfactant types and their respective performance results, which can guide the preparation of microemulsion systems in the field.

Because of the complex formation environment, a high cost is required for the preparation of a single surfactant system in order to obtain excellent and stable performance of microemulsions. Surfactant complexing can significantly improve surface activity, stability, temperature and salt resistance, and adsorption, compared to a single surfactant system, while also reducing costs. Currently, surfactant complex systems are widely used in the development of microemulsions in field experiments both domestically and internationally. The common types and effects of surfactant complexing are shown in the table below.

#### 2.4.2. Effect of the Choice of Cosurfactant on the Properties of Microemulsion

**Figure 6:**
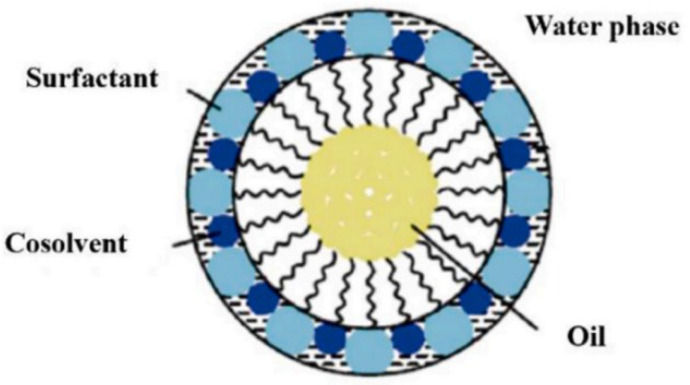
Position of cosurfactant molecules in a microemulsion [67].

In order to obtain stable microemulsions, the selection of cosurfactants is also crucial. Because they are soluble in both water and oil, they exhibit strong synergistic effects with surfactants. Therefore, suitable cosurfactants are generally chosen for the formulation of microemulsions [67]. The commonly used ones are short-chain alcohols, aldehydes, ethers, and their derivatives. In microemulsions, they primarily reduce interfacial tension, increase the fluidity of the interfacial film, adjust the hydrophilic–lipophilic balance (HLB) of surfactants, ensure the existence of aggregate structures, and disrupt liquid crystal or gel structures that hinder microemulsion formation, as shown in Figure 6. Additionally, in oilfield operations, cosurfactants with appropriate flash points should be selected according to reservoir temperatures to prevent the destruction of microemulsions in the reservoir.

#### 2.4.3. Effect of Oil Phase Selection on the Properties of Microemulsions

The commonly used ones are those that ensure no harm to reservoirs, environmental nonpollution, and biodegradability. According to the principle of similar solubility, the selection of the oil phase depends on whether the properties of the crude oil in the experimental area are similar to those of the selected oil phase. Moreover, the carbon chain length of the oil phase also influences the reduction in interfacial tension in microemulsions to some extent [59]. Additionally, the oil phase can be determined through HLB calculations. Common choices include biodiesel, kerosene, neem oil, d-limonene, and palm oil [68].

#### 2.4.4. Effect of Inorganic Salt Selection on the Properties of Microemulsion

Inorganic salts have minimal impact on the preparation of microemulsions, but variations in salt concentration can transition microemulsions from Winsor Type I to Winsor Type III. They also have a dual effect on ionic surfactants, as follows: reducing repulsion between their hydrophilic head groups and generating charged droplets during microemulsion formation [69].

In summary, when preparing the microemulsion, it is necessary to comprehensively consider various aspects, evaluate its economy, environmental protection, and applicability, and optimize the microemulsion components suitable for the target block.

#### 2.4.5. Studies on the Costs of Microemulsion Systems

Microemulsions, as complex systems with multiple components, require increased usage of each component in order to achieve better microemulsion phases, ultralow interfacial tension, and good stability, leading to an increase in costs. Because of the potential of microemulsion flooding to increase residual oil recovery after water flooding, researchers are now exploring ways to reduce the cost of microemulsions from different perspectives without compromising the performance of the system.

The previous discussion highlighted the excellent performance of nonionic and amphoteric surfactants but also emphasized their high cost. Currently, an important method for cost reduction involves adding appropriate anionic surfactants to nonionic/amphoteric surfactants, thereby reducing the amount of nonionic surfactants used. Additionally, anionic and nonionic/amphoteric surfactants can act synergistically, as shown in Table 3, to minimize precipitation, promote microemulsion formation to the fullest extent, and without compromising the system’s performance [70]. Wu et al. used a complex surfactant system consisting of sodium dodecylbenzene sulfonate and coconut oil fatty acid polyoxyethylene ether betaine in a mass ratio of 1:3 as the main surfactant for the microemulsion system. This reduced the amount of betaine used; however, under the corresponding conditions, the system formed a bicontinuous microemulsion, and the interfacial tension reached an extremely low level. The sand-packed model microemulsion flooding experiment resulted in a 23.25% increase in oil recovery [71]. Certainly, choosing natural materials to replace chemical products can also reduce the cost of surfactants. Badu et al. utilized castor oil to prepare a novel surfactant, sodium methyl ester sulfonate, which, when combined with cosurfactants and oil/water phases, formed a microemulsion. This approach yielded good economic results in oil displacement experiments [72]. As research progresses, some scholars have developed microemulsion formulations without surfactants and optimized the concentration of microemulsions to reduce costs [73].

In addition to the type of surfactants, the molecular structure of surfactants can influence the properties of microemulsions, accelerate the process of reaching optimal chemical formulations, and enhance crude oil recovery rates. Karasinghe et al. suggested that surfactants with super-short hydrophobic moieties, such as oleyl, tridecyl alcohol, and 2-ethylhexanol alkoxylatesm and their sulfonated and carboxymethylated derivatives, offer advantages such as short equilibrium times and low microemulsion viscosities. These chemicals can be commercially produced at low cost and remain economically viable even in the context of low oil prices [74]. Cosurfactants, as one component in a microemulsion system, can help reduce costs through the selection of suitable chemical agents. Jin et al. discovered intermediate products of isopropanol/n-butanol/ethanol during the microbial fermentation production process of n-butanol. These products not only reduce costs by around 10%, but also, when mixed with diesel, form monophasic microemulsions with small particle sizes and strong stability [75]. In addition, reducing the amount of the oil phase can also reduce costs. Lucas et al. developed a microemulsion system with low oil content, which serves as an economically efficient alternative to aromatic solvents. This system not only meets the increasing environmental requirements but also has the ability to repair formation damage and restore permeability [76].

### 2.5. Preparation Method of Microemulsion

The microemulsion is a homogeneous and transparent solution at the macro level, and an appropriate composition ratio can be spontaneously formed.

#### 2.5.1. Schulman Method

**Figure 7:**
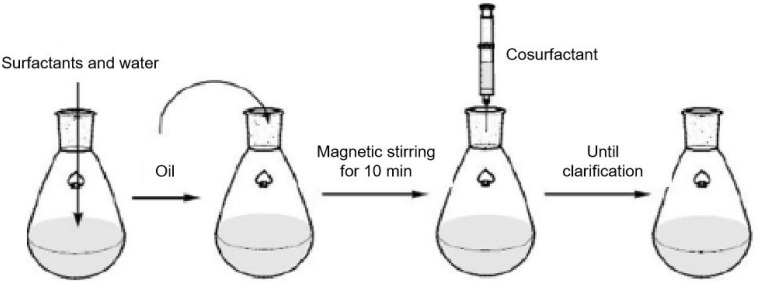
Preparation of a microemulsion by the Schulman method [77].

The Schulman method involves uniformly mixing organic solvents, water, and emulsifiers, followed by the gradual addition of alcohol to the emulsion. The operating procedure is illustrated in Figure 7. At a certain point, the system suddenly becomes transparent, thus forming a microemulsion. Different proportions of the components result in different types of microemulsions [77].

#### 2.5.2. Shah Method

**Figure 8:**
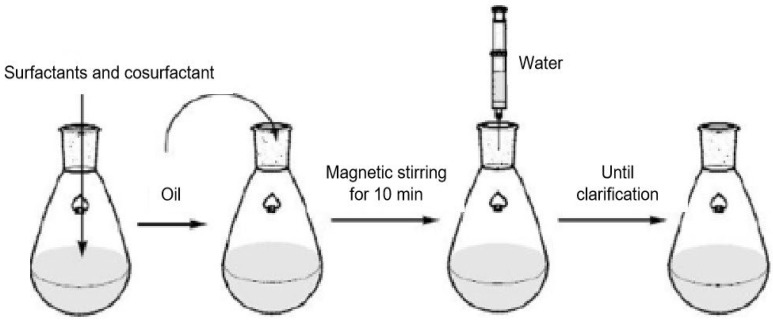
Preparation of a microemulsion by the Shah method [78].

The Shah method involves mixing oil, surfactant, and cosurfactant uniformly, followed by the addition of water (electrolyte aqueous solution) to the system. As shown in Figure 8. Within a certain range of ratios, the system becomes clear and transparent, forming a microemulsion [78].

In addition, methods such as salinity scanning, phase inversion temperature (PIT) method, supercritical fluid method, and membrane encapsulation method are employed to determine the optimal salinity and temperature for the system, resulting in the preparation of more stable and superior-performing microemulsion systems [79].

### 2.6. Characterization Method of Microemulsion

The characterization methods for microemulsions are primarily studied through phase behavior. However, there are other relevant techniques such as particle size analysis, polarized light microscopy, ζ-potential measurement, conductivity measurement, surface/interfacial tension analysis, and viscosity studies [80].

#### 2.6.1. Phase Behavior

**Figure 9:**
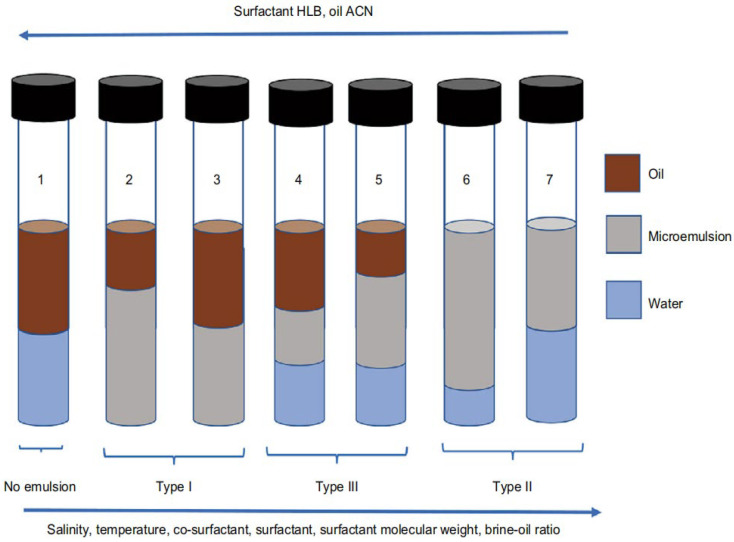
Microemulsion phase behavior of surfactants–water–oil as a function of different variables [81].

Through phase behavior testing, the behavior of reducing interfacial tension in microemulsions can be well described, thereby evaluating the feasibility of increasing oil recovery. The phase behavior of microemulsions is influenced by various factors, as shown in Figure 9. Studies on the phase behavior of microemulsions can be validated through pipette experiments, where micellar solutions are prepared in defined proportions and then poured into graduated glass pipettes. The oil is added in specific proportions, then the pipettes are sealed and gently shaken to ensure proper mixing. These pipettes are stored at the desired research temperature for several days until the mixture stabilizes and separates into phases. The volume grading of the cylinders aids in identifying the degree of microemulsion formation and the amount of dissolved oil/water solution. The dissolution parameters of water and oil are defined by the volume of water or oil dissolved in the microemulsion divided by the volume of surfactant present in the microemulsion, which is a key factor in economically designing microemulsions for enhanced oil recovery studies [81]. The method primarily used to study phase diagrams of microemulsions includes the Winsor phase diagram method [82,83], pseudo-ternary phase diagrams [84], and the fish-like phase diagram method [85].

#### 2.6.2. Particle Size Analysis

It is well known that there is an overlap between the particle sizes of microemulsions and nanoemulsions to some extent, and smaller particles exhibit higher mobility in porous media. To distinguish between them, their particle size distributions (PSDs) are observed. The main difference lies in the fact that the former is thermodynamically unstable, while the latter is thermodynamically stable. In other words, nanoemulsions can be considered conventional unstable emulsions composed of tiny particles [86]. Next, a particle size and distribution analysis can be performed through dynamic light scattering (DLS) experiments. As early as 1981, Nicoli et al. compared the correlation radius of water-in-oil microemulsions measured by DLS with the radius distribution of hexadecyltrimethylammonium bromide (CATB) micelles [87]. This experiment can provide insights into particle interactions, the dynamic particle size of microemulsions, and the possibility of micellar aggregation [88].

In addition to DLS, the particle size distribution in microemulsions can also be measured using a Zeta sizer, providing a more accurate particle size distribution [89]. Certainly, particle morphology and size distribution in microemulsions can also be directly observed through electron microscopy (SEM and TEM), providing high-resolution image information. SEM is suitable for observing surface morphology, while TEM is suitable for observing internal structure [90,91]. Although optical microscopy has relatively lower resolution, it can measure the particle size of microemulsions with larger diameters or irregular shapes through image analysis software [92].

#### 2.6.3. Interfacial Tension

**Figure 10:**
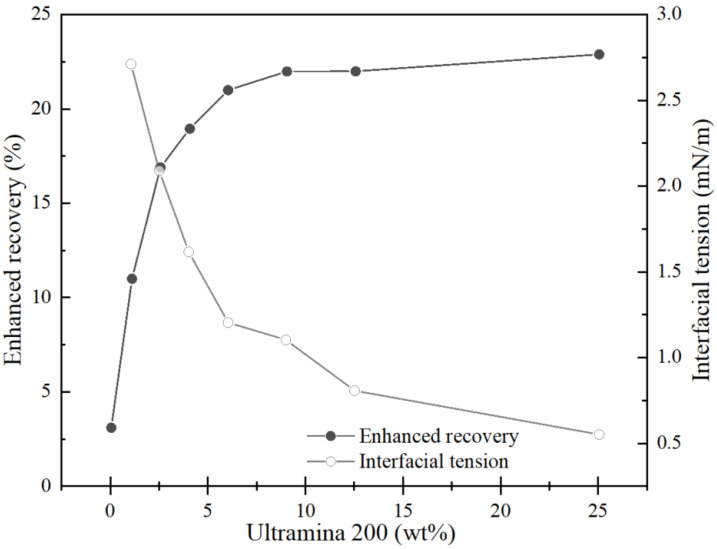
Relationship between interfacial tension and oil recovery [93].

In the process of enhancing oil recovery, the interfacial tension between oil and water is an important characteristic and is also one of the mechanisms through which microemulsions can significantly increase oil recovery rates [93]. Relevant studies have shown that the lower the interfacial tension, the greater the increase in oil recovery, as shown in Figure 10. The methods primarily used for determining interfacial tension are two-fold, as follows: the pendant drop method and the spinning drop method. In laboratory settings, the interfacial tension is commonly measured using a spinning drop tensiometer, while surface tension is determined using a surface tensiometer.

#### 2.6.4. Viscosity Test

Microemulsions with low apparent viscosity and high shear rates are easily injectable into reservoirs. Microemulsions in different systems exhibit varying viscosities, with cosurfactants in microemulsion systems playing a role in reducing apparent viscosity. The viscosity characteristics of microemulsions can be determined using a rheometer [94]. Microemulsion systems undergoing structural changes due to phase transitions can reflect variations in their viscosity. For instance, adding NaCl to microemulsions composed of anionic–nonionic surfactants enlarges droplet size, forming aggregates, and transitioning into a bicontinuous phase, which is also manifested in viscosity changes.

#### 2.6.5. Zeta Potential Test

The zeta potential primarily exists at the interface between solid surfaces and liquid media, affecting the charges present. A higher zeta potential is advantageous for microemulsion formation because increased repulsion prevents coalescence/aggregation, thereby enhancing stability and prolonging shelf life. Factors influencing zeta potential include salinity, temperature, pH, ionic strength, and surfactant concentration [95]. Therefore, it is necessary to determine the injection conditions of microemulsion based on the actual site conditions.

## 3. The Basic Method to Enhance Oil Recovery by Microemulsion

In tertiary oil recovery in oilfields, microemulsions are primarily utilized to enhance oil recovery through the following two methods: imbibition displacement and conventional displacement. These methods can operate independently or concurrently to boost oil recovery rates. Moreover, there are numerous factors that influence the effectiveness of both approaches in increasing oil recovery, which will be enumerated below.

### 3.1. Imbibition Displacement

For imbibition displacement, it typically refers to the replacement of nonwetting phase fluids by wetting phase fluids under the combined influence of interfacial tension, gravity, and capillary pressure differences among pores [96]. Imbibition displacement can be categorized into forward imbibition and reverse imbibition based on the magnitude of the Bond number (*NB*^−1^). The discriminant formula for *NB*^−1^ is as follows:

(3) $$NB^{-1}=\frac{C\sigma \sqrt{\frac{\Phi }{K}}}{\Delta \rho gh}$$

where *NB*^−1^ is the ratio of the capillary force to gravity; *c* is a constant related to the geometric size of the porous media; *σ* is the oil–water interfacial tension, mN/m; φ is the rock porosity; *k* is the rock permeability, mD; ∆ρ is the oil–water density difference, g/cm^3^; *g* is the acceleration of gravity, cm/s^2^; and *h* is the height of the rock, cm.

When *NB*^−1^ is less than 0.2, forward imbibition primarily occurs in the reservoir, with gravity playing the dominant role. When *NB*^−1^ is greater than 5, reverse imbibition occurs, where the imbibition process is mainly governed by capillary forces. If the value falls between these two, both flow mechanisms coexist in the porous media, with capillary forces and gravity jointly influencing the imbibition process [10].

Liu Weidong, Yao Tongyu et al. [97] improved Formula (3) by introducing the wettability parameter contact angle in their study on imbibition under different wetting conditions, resulting in Formula (4), as follows:

(4) $$NB^{-1}=2\frac{C\sigma \cos\theta \sqrt{\frac{\Phi }{K}}}{\Delta \rho gh}$$

For imbibition displacement, there are many factors affecting enhanced oil recovery, including the following several important factors.

#### 3.1.1. Influence of Rock Wettability

The wettability of rocks is the most critical factor influencing imbibition efficiency, and based on the magnitude of the wetting contact angle, wettability is classified as oil-wet, water-wet, or neutral wetting. Morrow conducted experiments on the influence of wettability on imbibition efficiency and found significant differences in the imbibition recovery rates of cores with different wettability. When the rock surface is hydrophilic, capillary forces are prominent, resulting in higher imbibition recovery rates [98]. Zhao et al. conducted wettability tests on microemulsions at different concentrations, and the experiments indicated that with increasing concentration, the microemulsion’s wettability improvement effect enhanced. When the concentration reached 0.3%, it reduced the contact angle from 130.6° to 11.7°. Under this wettability condition, the imbibition efficiency could reach 43.2% [99].

#### 3.1.2. Influence of Oil–Water Interfacial Tension

Interfacial tension plays a crucial role in the imbibition process of microemulsions, and the capillary number is an essential parameter characterizing the imbibition process. The higher the capillary number, the greater the imbibition efficiency, resulting in better reduction in residual oil saturation. Capillary number is inversely proportional to the interfacial tension between oil and water and directly proportional to the imbibition rate and viscosity coefficient. Hence, lower interfacial tension leads to higher capillary numbers and better imbibition effects. In water-wet reservoirs, interfacial tension provides spontaneous imbibition force, facilitating crude oil deformation, weakening the Jamin effect, and reducing flow resistance, thereby enhancing the effectiveness of imbibition displacement of crude oil [100]. Reducing interfacial tension can decrease adhesion energy and enhance oil washing efficiency. Xiao et al. evaluated the interfacial tension of microemulsion systems at different concentrations and conducted dynamic and static imbibition experiments on microemulsions with varying interfacial tension systems. The results indicate that ultralow interfacial tension can reduce the adhesion energy of the oil phase, facilitating the detachment and deformation of crude oil, thereby improving imbibition efficiency [101]. Attar et al. discovered through experiments that the final recovery and imbibition rate of spontaneous imbibition are significantly correlated with the interfacial tension between oil and imbibing fluid. The lower the interfacial tension, the higher the recovery rate obtained [102].

However, the reduction in interfacial tension also leads to a decrease in capillary pressure. In low-permeability to tight reservoirs, where pore throats are small, capillary forces are the primary driving force. The decrease in interfacial tension results in a reduction in this driving force, leading to a decline in imbibition recovery rates. Currently, an increasing number of researchers believe that there is often an optimal interfacial tension for imbibition systems, maximizing imbibition efficiency. Qin et al. conducted static imbibition experiments on RFFF solutions with different interfacial tensions. The results indicate that the imbibition efficiency is highest when the interfacial tension is 0.1 mN/m. Lower or higher interfacial tensions do not improve the imbibition recovery rate [103]. Therefore, in the process of enhancing imbibition recovery, one should not blindly pursue excessively high or low interfacial tensions. Specific analysis should be conducted on the basis of the physical properties of the target reservoir.

#### 3.1.3. Influence of the Core Pore Size

The size of rock pores affects both the permeability and porosity of the rock cores, which are intrinsic properties of reservoirs and determine their quality. Generally, larger porosity corresponds to higher permeability, indicating better reservoir connectivity. Yang Xue et al. utilized nuclear magnetic resonance technology and high-pressure mercury injection tests to study the micropore structure of rock cores. They designed spontaneous imbibition experiments simulating reservoir conditions. The results revealed that tight rock cores have three types of pore structures, with the ultra-micropores being the primary storage space for crude oil, accounting for nearly 80% of the pore volume. Ultra-micropores have small pore diameters and strong capillary forces, resulting in the highest imbibition efficiency during the initial stages of imbibition. They also contribute the most to the recovery rate of imbibed crude oil. After simulating fracturing, the overall imbibition recovery rate of the fractured rock cores increased by 24.7% [104]. Cheng evaluated the contribution of different pores within rock cores to imbibition recovery rates through nuclear magnetic resonance experiments. The results showed that micropores and small pores contributed the most to imbibition recovery rates, and rock cores treated with deionized water to alter their wetting state exhibited better imbibition recovery rate effects [105]. This also indicates that in low-permeability reservoirs, imbibition displacement is an effective method for enhancing oil recovery.

Imbibition displacement in reservoirs is a complex process [9]. In addition to the aforementioned influencing factors, other factors affecting imbibition include temperature, pressure, viscosity, and water saturation. Microemulsions exhibit excellent performance and can enhance imbibition displacement effects by reducing the oil–water interfacial tension and altering rock wettability.

### 3.2. Conventional Displacement Replacement

Microemulsions, in addition to imbibition displacement, can also serve as displacement agents, entering the formation in a manner similar to miscible flooding, utilizing the permeation process to displace crude oil and, thereby, enhance oil recovery. Earlier, we have discussed the small particle size and low interfacial tension characteristics of microemulsions. Taking advantage of these traits, microemulsions can be utilized in low-permeability/tight reservoirs. However, displacement effectiveness is constrained by various factors, with several being particularly significant.

#### 3.2.1. Influence of Microscopic Pore Structure

The size of micropore structures primarily determines the injectability of microemulsions. In actual displacement experiments, the compatibility between formation pores and microemulsion particle size is a primary consideration. Zhao Hongpeng et al. measured the particle size distribution of carboxymethyl betaine microemulsion systems using a laser particle size analyzer, which fell within the range of 0.01–0.02 μm, closely resembling a normal distribution. Through analysis of microscopic residual oil saturation, they found that the proportion of pores containing residual oil decreased with decreasing pore radius. Smaller microemulsion particle sizes facilitate their entry into pores for displacing residual oil. For the same pore radius, smaller microemulsion particle sizes result in a smaller proportion of pores containing residual oil [106]. Zhao Mi et al. studied the in situ injection of petroleum sulfonate to form microemulsions with crude oil, analyzing the changes in particle size during the solubilization process. The results showed that the microemulsion particle size continuously changed over time. Initially, the dispersed phase particle size increased significantly upon contact, followed by a decrease in the effective particle size, half-width, and polydispersity index [107]. This indicates enhanced injectability.

#### 3.2.2. Influence of Displacement Speed

**Figure 11:**
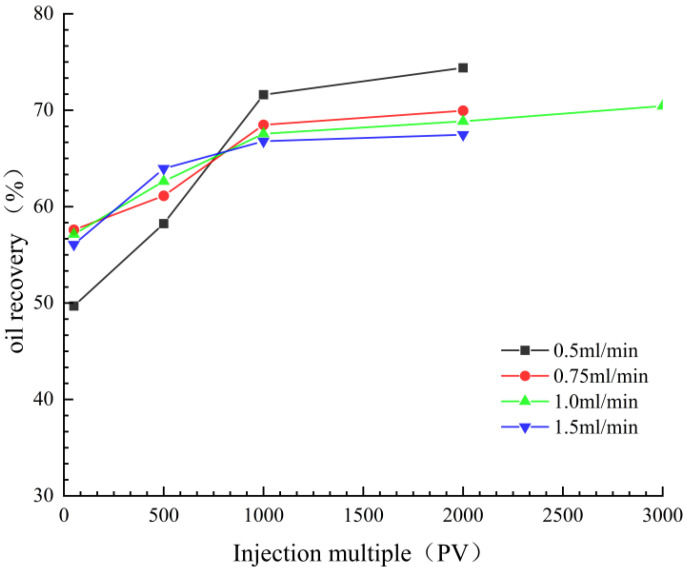
Relationship between displacement velocity and oil recovery [108].

After prolonged water flushing, the micropore characteristics of reservoirs undergo significant changes, necessitating adjustments in displacement velocity for further efficient development. Ma Kuiqian et al. studied the impact of different displacement velocities on recovery rates, as illustrated in Figure 11. It was found that under various displacement velocities, there exists an optimal displacement velocity leading to the highest recovery rate [108].

#### 3.2.3. Net Burden Pressure (Confining Pressure)

During water flooding experiments, the rock core holder applies a certain confining pressure to simulate the actual net overburden pressure in the formation. The net overburden pressure directly affects the size of micropores, and in deep, high-pressure, low-permeability reservoirs, significant net overburden pressure can cause changes in pore structure during displacement. Analysis reveals that as the net overburden pressure increases, throat size decreases, the proportion of ineffective throats increases, and the proportion of immobile oil in the pore-throat system also rises. Consequently, the oil displacement efficiency and water-free recovery rate decrease accordingly. The greater the net overburden pressure (and, thus, the greater the net overburden pressure deficit), the poorer the mobility of the crude oil [109].

Like the imbibition displacement process, displacement experiments are also complex processes involving two-phase flow of oil and water. In addition to the aforementioned constraining factors, displacement experiments are also influenced by factors such as displacement agent concentration, temperature, and oil–water saturation.

## 4. Application of Microemulsion in Enhanced Oil Recovery

As oilfield development progresses and the need to increase oil recovery becomes more pressing, various oil recovery methods are widely utilized. These methods each have their unique mechanisms, technical characteristics, advantages, disadvantages, and research progress. In order to better understand their differences and applicability, a brief comparison of these oil recovery methods will be presented below, and the comparative results are presented in Table 4.

Compared to other oil recovery methods, microemulsion flooding has shown a greater increase in oil recovery rates, resulting in reduced chemical costs per ton of oil. Furthermore, with the maturation of chemical production technology, the prices of the numerous components of microemulsions can also be reduced.

Based on the unique and outstanding performance of microemulsions summarized above, including ultralow interfacial tension, small droplet size, biphasic wetting, high surface activity, increased scanning volume of micro-nanopores, enhanced permeability, and displacement efficiency of micro-nanopore matrix, as well as significantly reduced interfacial tension between oil and water [110], this section is divided into the following two parts: laboratory preparation of microemulsion formulations and their effectiveness in enhancing recovery, as well as the application of microemulsions in oilfield operations.

### 4.1. Microemulsion Indoor Enhanced Oil Recovery Effect

Currently, laboratories worldwide have made significant progress in the research of microemulsion formulations, preparing microemulsion systems suitable for various types of oilfields. Kumar et al. prepared a microemulsion using Tween 40 as the main surfactant for sandstone reservoirs, resulting in a 26.4% increase in recovery compared to water flooding [111]. Dantas et al. investigated the impact of acid flow on carbonate reservoirs and its effect on oil recovery operations, preparing acidic microemulsions. Oil displacement experiments showed that acidic microemulsions in porous media can delay the dissolution reaction of rocks in hydrochloric acid, avoiding the formation of high-permeability channels, ultimately achieving an additional 30% recovery [112]. Aum et al. prepared acid microemulsions containing HCl using the nonionic surfactant ALKL90 as the surfactant, demonstrating that under acidic conditions, the stability of microemulsions in carbonate formations is greatly enhanced, allowing microemulsions to effectively promote oil droplet movement [113]. Dantas et al. prepared an alkaline–polymer microemulsion system containing polymer HPAM to evaluate the interaction mechanism between rock and microemulsions in Botucatu sandstone. They demonstrated the oil displacement ability at low concentrations and excellent wettability alteration efficiency. The addition of polymer increased the system’s viscosity, resulting in a recovery rate of around 30% [114]. To address the lack of evaluation of microemulsion displacement in high-temperature reservoirs, Karambeigi et al. spontaneously formed microemulsions using components such as polyoxyethylene sorbitan monooleate (Tween 80) and biodiesel at 75 °C. They characterized the interfacial properties and wettability alteration abilities, conducting three imbibition experiments which yielded a recovery rate of approximately 20% [115]. The selection of the oil phase in this system opens up a new path for renewable and environmentally friendly microemulsion formulations. Nourafkan et al. studied the oil displacement ability of stable water-in-oil microemulsions with added magnetic iron oxide and titanium dioxide nanoparticles in harsh high-temperature and high-salinity environments. Oil displacement tests confirmed an increase in recovery rates with the addition of nanoparticles. This improvement was mainly due to the enhanced stability of the microemulsion system with the inclusion of nanoparticles, as well as the improvement in the shear thinning characteristics of the microemulsion. The highest recovery rate of 77% was achieved in nanoparticle flow control chip experiments [116]. Zhao Baiyang et al. used an orthogonal method to screen a microemulsion system with 0.3% IOS (internal olefin sulfonate) for field application in the low-permeability Yushulin oilfield in Daqing. This system was able to form an ultralow interfacial tension with the crude oil, reaching 6 × 10^−4^ mN/m. Additionally, the particle size distribution of the system matches the diameter of the rock core throats, facilitating the rapid entry of the microemulsion into the formation throats. This system effectively displaced the residual oil in the low-permeability reservoir after water flooding [117]. Yin Daiyin et al. selected a compounded surfactant microemulsion system, combining ANG7-IV-7 with alkyl benzene sulfonate in a 4:1 ratio, using Daqing crude oil as the oil phase. This system exhibited excellent temperature and salt resistance and strong resistance to calcium and magnesium ions, without undergoing chromatographic separation. After compounding, the interfacial tension decreased to below 10^−4^ mN/m, resulting in a 12.8% increase in recovery rate in low-permeability rock cores after water flooding [118]. Song Hongzhi et al. pioneered the use of microemulsions to enhance oil recovery in offshore heavy oilfields, preparing a stable low interfacial tension microemulsion system. The synergistic action of the components in this system increased the recovery rate by 30%, and it does not require backflushing during use, effectively addressing issues such as near-wellbore plugging and high wellbore pressure caused by injection difficulties [119]. Lv qichao et al. selected a microemulsion system composed of 1.1% Tween-40 and 1.2% sulfate-type anionic surfactant as the primary surfactants. They added 6.0% cosurfactant and 2.5% sodium chloride to form the microemulsion system, which could enhance the solubilization of residual oil at the injection end in highly water-bearing porous media (with a water content of 95%). Simultaneously, during the injection process, the system utilized the elasticity and mechanical properties to affect and squeeze the residual oil downstream. Under the action of ultralow interfacial tension, it facilitated the connection of scattered residual oil regions, thereby increasing the recovery rate [120]. With the rise of nanotechnology, Qin et al. considered the influence of nanoparticles on the performance of microemulsions. Compared to traditional microemulsions, nanoparticles synergistically interact with microemulsions; microemulsions emulsify oil droplets into smaller ones, thereby enhancing the recovery rate [121]. nanoparticles embed into the contact surface between rocks and oil droplets, generating detachment pressure, leading to the detachment of oil droplets [122].

The following Table 5 lists some microemulsion formulations that have shown excellent performance in laboratory experiments.

### 4.2. Effect of Microemulsion on Oilfield Field Production Increase

Oilfields worldwide are actively conducting pioneering experiments on the use of microemulsions to enhance oil recovery in field operations. The Robinson oilfield in the United States conducted experiments on microemulsion-enhanced oil recovery after polymer flooding, achieving significant improvements in oil displacement efficiency with a 30% increase in recovery rates [8]. Meanwhile, relevant field experiments have also been conducted in Canada, France, Japan, and other locations, all of which have yielded positive results. In Nigeria’s Rona conventional sandstone oilfield, microemulsions were used to treat the low-producing wells Rona-07S and Rona-12S, repairing the damaged near-wellbore area and restoring production. Compared to the initial production levels, the production in these two areas increased by 1164 barrels per day and 205 barrels per day, respectively [128]. The French National Oil Company conducted pioneering experiments on microemulsions in the Chateaurenard oilfield in the southern part of the Paris Basin. In this area, the efficiency of water flooding prior to microemulsion treatment was poor, with an average water content reaching 88%. After treatment, the oil recovery efficiency increased from the original 3.2 m^3^/d to 12 m^3^/d, nearly quadrupling the production rate [129]. In 2006, the Tin Fouyé oilfield in In Amenas, Algeria, had a water cut exceeding 90%, yet approximately 43% of the original oil in place remained in the reservoir. By employing microemulsion injection, the oil recovery rate in this field saw a significant increase [130]. In major oilfields in China, efforts to increase oil recovery focus on techniques such as pressure reduction and water injection, restoring relative permeability, and improving near-wellbore plugging. In 1990, a single-well microemulsion flooding experiment was conducted in well F-184 of the Laojunmiao oilfield. The injection improved injectivity, significantly reduced injection pressure, and markedly improved reservoir permeability. The water content near the central well decreased from 99.5% to 86.5%, resulting in a total oil increase of 142 t [131]. In 2003, Li Fuyou et al. developed a self-made microemulsion system based on the Liaohe oilfield. This system exhibited stable performance and showed significant benefits in alleviating blockages in heavy oilfields. They conducted six oil displacement experiments at the Gao Sheng oil production plant, resulting in a cumulative oil increase of 842.3 t across the six wells [132]. In recent years, with the development of the Zhuangxi oilfield, the reservoir properties have become increasingly complex, and the well pattern deployment has been challenged, leading to a gradual increase in water injection pressure. Following microemulsion treatment, there has been a noticeable reduction in pressure and an increase in water injection, with an average single well pressure reduction of 7.2 MPa and an increase of 7117 m^3^. This has resulted in a cumulative oil increase of 3152 t, effectively addressing the difficulties in water injection for low and ultralow permeability reservoirs [133]. In 2020, the Jidong oilfield conducted on-site experiments injecting microemulsions to achieve pressure reduction and enhanced oil recovery. Following acidification, a 0.5% microemulsion solution was injected at a volume of 80–100 m^3^. The experimental results showed an increase in reservoir water phase permeability, with a pressure reduction of 8 MPa in a single well and a cumulative increase in injection of 3.5 × 10^4^ m^3^ across the well group. After a half-month shut-in period, the total cumulative oil production increased by 3000 t [134].

## 5. Micro-Remaining Oil Characterization Method of Microemulsion Flooding

The mechanism, quantity, and spatial distribution of microscopic residual oil in pores are important research topics for evaluating reservoir oil content and enhancing oil recovery at different stages of reservoir development. Because of the small size of microscopic residual oil and the complexity of influencing factors, special methods are required for research [135]. Currently, commonly used methods include CT scanning technology [136], scanning electron microscopy (SEM) technology [137], nuclear magnetic resonance imaging technology [138], and microscopic glass-etching models [139].

### 5.1. Micro-Etching Glass Model

**Figure 12:**
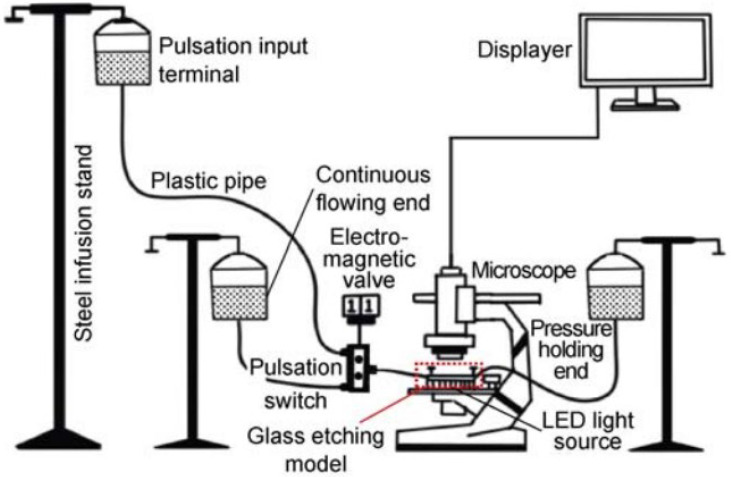
Microvisualization device [143].

The etched-glass method is a conventional approach for creating microscopic models. As shown in Figure 12, this method utilizes photolithography to simulate reservoir pore throats and prepare them as microscopic, visually accessible etched models. These models are widely used to study the distribution of residual oil in the reservoir. Zhou et al. utilized microscopic models to discover that the viscoelastic properties of polymers play a crucial role in enhancing the recovery efficiency of blind-end residual oil [140]. Maaref et al. conducted measurements on the recovery rate of emulsion flooding under varying salinity levels and concluded that the higher the salinity of the brine, the lower the recovery rate [141]. Wu et al. utilized microscopic visualization models to study the mechanism of foam flooding on heavy oil in porous media, providing theoretical support for foam flooding production [142]. Through microscopic experiments, the distribution, morphology, and migration process of residual oil can be directly observed, facilitating the analysis of the mechanism of residual oil formation.

### 5.2. Magnetic Resonance Imaging Technology (MRI)

**Figure 13:**
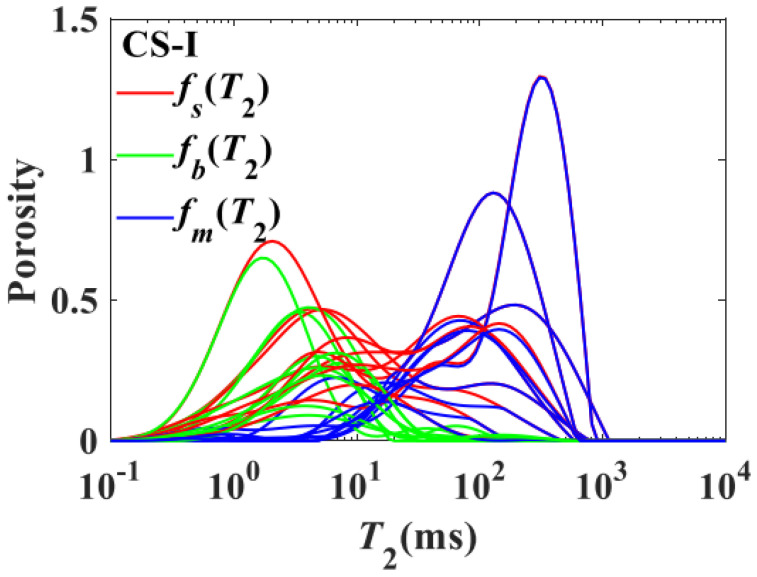
Nuclear magnetic resonance T_2_ spectrum [146].

The nuclear magnetic resonance (NMR) technique primarily utilizes the physical properties of hydrogen nuclei to obtain well-defined pulsed NMR measurement signals, offering the unique advantage of nondestructive sample detection. The advantage of one-dimensional T_2_ spectrum lies in its capability to measure the oil or water content inside the pores of a core saturated with a single fluid. However, when oil and water coexist in the core, the obtained T_2_ spectrum results in the superposition of oil and water signal values, making it challenging to accurately differentiate between the two. This limitation presents constraints on the study of microscopic residual oil. However, the interference of water signals can be eliminated to some extent by using manganese water during the core saturation process, thereby alleviating this limitation [138]. Xiong et al. used low-field nuclear magnetic resonance to obtain high-resolution single-oil signals below the water freezing point, achieving dual oil and water signals at room temperature. This enabled the determination of oil and water saturation, and by calculating the oil and water saturation distribution in pores of different sizes through the conversion of nuclear magnetic resonance transverse relaxation time [144]. Zhang et al. used the T_2_ spectrum of nuclear magnetic resonance technology and the adsorption ratio theoretical model to clearly describe the microscopic distribution of adsorbed oil and free oil, demonstrating that adsorbed oil mainly concentrates in micropores, while free oil mainly accumulates in medium to large pores. The microscopic pore structure influences the microscopic distribution characteristics of the shale oil [145]. Jin et al. utilized nuclear magnetic resonance (NMR) measurements to quantitatively characterize the distribution of bound and mobile fluids in porous rocks. They conducted NMR experiments on 19 conventional sandstone samples from the Songliao Basin and 19 tight sandstone samples from the Ordos Basin under conditions of complete saturation with water and bound water, obtaining the transverse relaxation time (T_2_) distributions of saturated water, bound water, and mobile water [146], as shown in Figure 13.

### 5.3. CT Scan Technique

CT scanning technology can not only reconstruct images of the microscopic residual oil distribution at different displacement times for various displacement fluids, but can also calculate the porosity and oil saturation parameters of the rock core by converting X-ray attenuation coefficients into CT values. This allows for quantitative analysis of the heterogeneity of experimental rock cores and the microscopic residual oil at different times during each displacement process [147]. Zhang utilized CT scanning technology to observe the distribution of residual oil after water flooding, and found that throughout the entire water flooding process, the cluster flow state transitioned between continuous displacement and fragmentation into discontinuous phases [148].

CT scanning technology can not only reconstruct the microscopic remaining oil distribution images of different displacement fluids at different displacement times but also convert the X-ray attenuation coefficient into the CT number. It can also calculate the porosity and oil saturation parameters of the core, so as to quantitatively analyze the heterogeneity of the experimental core and the microscopic remaining oil at different displacement times in each displacement process [147]. Zhang used CT scanning technology to observe the distribution of remaining oil after water flooding and found that during the whole water flooding process, the cluster flow state was continuously displaced or broken into discontinuous phases [148]. Iglauer studied the effect of wettability on the size of remaining oil clusters by CT and concluded that the remaining oil tends to be distributed in the center of larger pores after water wetting, while the remaining oil in large clusters after oil wetting is less [149]. However, when CT technology is used in tight reservoirs with small pore radius, it is greatly affected by resolution, and it is difficult to quantitatively describe the change in the remaining oil at this time. Iglauer studied the influence of wettability on the size of residual oil clusters using CT and proposed that after water wetting, residual oil tends to be distributed at the center of larger pores, while after oil wetting, less residual oil is found in larger clusters. However, when CT technology is applied to tight oil reservoirs with small pore radii, it is significantly affected by resolution, making it challenging to quantitatively describe the changes in residual oil at this scale [149].

## 6. Summary, Challenges, and Development Trends

After conducting comprehensive research on the component selection, formation mechanism, characteristics of use, and application in enhancing crude oil recovery of microemulsions, a systematic summary was made regarding microemulsion systems, the challenges they face, and their future development trends.

### 6.1. Summary

As oilfield development progresses deeper and geological conditions evolve, the demand for enhanced oil recovery becomes increasingly pressing. Researchers and institutions are increasingly focusing on and investing in microemulsion flooding. Citation reports on microemulsions from “Web of Science” website show an explosive increase starting from 2002, peaking in 2020. These trends indicate the excellent performance of microemulsions in various aspects and their ability to effectively enhance reservoir modification outcomes.

Microemulsions, composed of surfactants, cosurfactants, oil phase, and aqueous phase (including inorganic salts), exhibit particle sizes ranging from 1 to 100 nanometers. They play a significant role in enhancing the recovery of crude oil in low-permeability oil reservoirs following water flooding or polymer flooding, due to their ultralow interfacial tension and strong solubilization capabilities. Variations in the formulation of microemulsions can lead to changes in their microstructure and macroscopic phase behavior, thereby resulting in performance differences among different systems.

### 6.2. Challenge

(1) As tertiary oil recovery operations progress, the reservoir environment becomes increasingly harsh, particularly in high-temperature and high-salinity oilfields where the quality of crude oil deteriorates. The existing microemulsion formulations are no longer suitable for these conditions. It is necessary to identify and develop new formulations that can adapt to the changing characteristics of the reservoir environment to maintain and enhance oil recovery efficiency.
(2) The formulation of microemulsions commonly employs short-chain alcohols as cosurfactants, which typically have low flash points (generally below 60 °C). This characteristic can be inadequate for the current reservoir conditions, where the formation temperatures are often higher than 60 °C, potentially leading to safety incidents.
(3) The application of microemulsions in the field exhibits a lack of universality; the same formulation, when utilized in different oil reservoirs, may yield varying degrees of enhanced oil recovery (EOR) effectiveness. Concurrently, there is significant debate regarding whether microemulsions should be prepared ex situ and then injected into the subsurface (non-in situ injection), or whether the individual components, such as surfactants, should be injected into the reservoir where they interact with the crude oil to form microemulsions in situ (in situ injection).
(4) The efficacy of microemulsions in oil reservoirs with varying permeabilities is not yet well-defined, and there is a need for further in-depth investigation into the mechanisms of crude oil mobilization and the migration patterns within such formations.

### 6.3. Development Trends

The future development trend of microemulsion mainly has the following four points:

(1) Transitioning from microemulsion systems based on single surfactants to those employing blended surfactants in research can lead to a reduction in the chemical cost per ton of oil. This approach also aims to enhance the fundamental properties of microemulsions, such as their thermal and salinity resistance, as well as their ability to withstand shear forces.
(2) The consolidation of numerous existing performance evaluation methods is essential to establish new standards or criteria for the assessment of microemulsion performance.
(3) By integrating with the field of molecular simulation, the study of the interaction mechanisms between microemulsions and various rock formations can be conducted.
(4) At present, research often focuses on either the macroscopic phase behavior or the microscopic structure of microemulsions in isolation. Moving forward, it is of paramount importance to investigate the impact mechanisms of different factors on the phase behavior of microemulsions by combining the study of their macroscopic phase states with their microscopic structures. This integrated approach will provide a more comprehensive understanding of the complex interactions and behaviors of microemulsions in various conditions.

## Figures and Tables

**Table 2:** Effect of Microemulsion Prepared by Different Types of Surfactants.

Type	The Surfactants in the Microemulsion Formulation	Researchers	Results
Anionic surfactants	Sodium dodecylbenzenesulfonate	Zhao Xuezhi [54]	In situ injection forms the following two types of emulsions: smaller droplets enhance the fluidity of oil, while larger droplets block the main pathways, improving sweep efficiency. Compared to water flooding, the recovery rate can be increased by 30%.
		Yuan Ying [55]	The identification of a single inorganic salt within the sodium dodecylbenzene sulfonate system predicted the mixed salt effect, providing a new method to simplify mixed salt microemulsion systems.
		Ali Rezaie [56]	Accurately assessing the influence of each salt on the phase behavior of microemulsions, the optimal salinity and solubilization parameters for different types of salts are consistent with Hofmeister series.
	Sodium dodecyl sulfate	Zamula YS [57]	Through atomic force microscopy, the microscopic structure of microemulsions was studied. By comparing differences in surface morphology, single particles or complex structures with well-defined shapes corresponded to the bicontinuous structure of microemulsions.
		Pal N [58]	Through physical-chemical evaluation studies to assess the suitability of microemulsions for consistency improvement techniques, the potential of the formulation’s use was demonstrated.
	Fatty Acid Methyl Ester Sulfonate	Pal N [59]	The interfacial tension values between microemulsion systems and hydrocarbon systems were found to be significantly lower than the interfacial tension values between surfactant and hydrocarbon systems. After injection of this system, the recovery rate was close to around 30% of conventional secondary water flooding.
Nonionic Surfactants	Tergitol	Achinta Bera [60]	The interfacial tension between oil and the microemulsion phase is a strong function of surfactant concentration and salinity.
	Polyoxyethylene ether	Zhang Haixia [61]	Longer chain length and fewer ethylene oxide units in long-chain polyoxyethylene ether surfactants make it easier to form W/O microemulsions. In this case, microemulsions exhibit excellent high-temperature and low-temperature stability, as well as lower interfacial tension.
	Pentaethylene glycol monododecyl ether	Schmidt RF [62]	Compared to the ionic surfactant AOT, Pentaethylene glycol monododecyl ether is best described using a symmetrically disordered lamellar model.
	Fatty alcohol polyoxyethylene ether 9 (AEO 9)	Xu H [63]	It is capable of forming stable microemulsions, and the oil phase in this system can control the release of adhered surfactants. Additionally, there exists a competitive adsorption relationship between the oil phase and the rock surface for free surfactants in the solution, which offers the potential to reduce surfactant adsorption losses and extend the penetration distance of surfactants into the reservoir matrix.
Cationic surfactants	Cetyltrimethylammonium bromide	Maria LDO [64]	Improving the migration rate of nanoparticles in porous limestone media and their stability in saline solutions containing high concentrations of divalent cations, oil displacement tests were conducted in uncemented porous media, achieving a recovery rate of up to 60%.
	Imidazoliumbased ionic liquid	Fattahi R [65]	The addition of nanoparticles to cationic surfactants enables the formation of stable microemulsions, with the ability to reduce interfacial tension similar to microemulsions formed by anionic surfactants.
	Benzethonium chloride	Phaodee P [66]	It can promote the formation of the middle phase in microemulsions when copolymerized with different anionic surfactants, while also reducing or eliminating the demand for electrolytes, enhancing solubilization or cosolvency.

**Table 3:** Comparison of the properties of different emulsions.

Compound Category	Property
Homoelectric surfactant compounding	For the compounding of homologous surfactants, the performance is among various components. Different types of compounding also have synergistic effects (anionic-anionic, nonionic-nonionic).
Anion + cation	It has the strongest synergistic effect, but it is easy to precipitate in aqueous solution, so it needs to be designed reasonably.
Anion + nonion	It can reduce the repulsion of the hydrophilic head group, increase the surface activity, and make up for the shortcomings of nonionic temperature resistance and anion salt resistance.
Cationic + nonionic	There is a synergistic effect, but the effect is not as good as that of anionic-nonionic compound (the oxygen atom on the ethoxy group of the nonionic surfactant can be combined with water by hydrogen bond, which is positively charged and repelled with the cationic surfactant).

**Table 4:** Comparison of different oil displacement methods.

Displacement Methods	Mechanism	Technical Characteristics	Advantage	Disadvantage	Research Progress
Polymer flooding (P)	Increase the sweep coefficient	Increase water phase viscosity and improve fluidity ratio	Good oil increasing effect, cost saving, and simple operation	Narrow applicability and low polymer stability	industrial application
Surfactant flooding (S)	Improve oil washing efficiency and increase sweep coefficient	Reduce the interfacial tension between oil and water; changing the surface wettability of porous media	Significant effect and convenient use	High cost, difficult to treat produced liquid	Pilot Test
Alkaline waterflooding (A)	Same as above	Reduce the interfacial tension between oil and water; changing the surface wettability of porous media	Low-cost and simple operation	Corroded pipelines, scaling, and difficult to treat produced liquids	Pilot Test
Ternary composite flooding (ASP)	Same as above	Improving the fluidity ratio and altering the surface wettability of porous media	The advantages of combining polymers and surfactants	High cost, complex operation, corrosion of pipelines, scaling	field test
Binary compound flooding (SP)	Same as above	The increase in oil recovery rate is significant and has a wide range of applications	Similar to ternary composite flooding	High cost and complex operation	field test
Gas flooding	Same as above	Reduce crude oil viscosity, eliminate Jamin effect, reduce interfacial tension and capillary pressure	Suitable for various reservoir conditions, with a significant increase in oil recovery rate	High requirements for ground gas injection systems and equipment, high injection pressure, and difficulty in continuous injection	Continuously optimizing and improving in practical applications
Nano chemical flooding	Same as above	Utilizing the special properties of nanomaterials	Expected to significantly increase oil recovery rate	Currently still in the interior research stage	Laboratory Study
Microemulsion flooding	Same as above	Ultralow oil–water interfacial tension; changing the surface wettability of porous media; strong solubilizing oil and emulsifying ability	Expected to maximize oil recovery	The system has multiple components and high costs	Continuously optimizing and improving in practical applications

**Table 5:** Laboratory microemulsion formulations.

Researchers	Formulation
Xu [123]	4 wt% Sodium dodecyl sulfate + 8 wt%n-butanol + Pentane + 6%KCl
Zhao [124]	0.5 wt% Internal olefin sulfonate + 3.5%Sodium dodecyl sulfate + 5 wt%2-butanol + 1-dodecanol + 6.5 wt%NaCl
Oliveira [125]	Nonylphenol ethoxylate 100 + n-butanol + kerosene + Synthesis of Produced Water
Hon [126]	Sodium C14-16 olefin sulfonate + intermediate oil + NaCl
Lu [127]	Ethylene glycol + decane + water

## Data Availability

Data are contained within the article.
